# Natural Antioxidant-Isoliquiritigenin Ameliorates Contractile Dysfunction of Hypoxic Cardiomyocytes via AMPK Signaling Pathway

**DOI:** 10.1155/2013/390890

**Published:** 2013-09-16

**Authors:** Xiaoyu Zhang, Ping Zhu, Xiuying Zhang, Yina Ma, Wenguang Li, Ji-Mei Chen, Hui-Ming Guo, Richard Bucala, Jian Zhuang, Ji Li

**Affiliations:** ^1^Institute of Physiology, School of Basic Medicine Sciences, Lanzhou University, Lanzhou 730000, China; ^2^Department of Pharmacology and Toxicology, School of Medicine and Biomedical Sciences, University at Buffalo-SUNY University of New York, Buffalo, NY 14214, USA; ^3^Department of Cardiovascular Surgery, Guangdong Cardiovascular Institute, Guangdong General Hospital, Guangdong Academy of Medical Sciences, Guangzhou 510080, China; ^4^Department of Emergency, Gansu Provincial Hospital, Lanzhou 730000, China; ^5^Department of Internal Medicine, Yale University School of Medicine, New Haven, CT 06520, USA

## Abstract

Isoliquiritigenin (ISL), a simple chalcone-type flavonoid, is derived from licorice compounds and is mainly present in foods, beverages, and tobacco. Reactive oxygen species (ROS) is a critical factor involved in modulating cardiac stress response signaling during ischemia and reperfusion. We hypothesize that ISL as a natural antioxidant may protect heart against ischemic injury via modulating cellular redox status and regulating cardioprotective signaling pathways. The fluorescent probe H_2_DCFDA was used to measure the level of intracellular ROS. The glucose uptake was determined by 2-deoxy-D-glucose-^3^H accumulation. The IonOptix System measured the contractile function of isolated cardiomyocytes. The results demonstrated that ISL treatment markedly ameliorated cardiomyocytes contractile dysfunction caused by hypoxia. ISL significantly stimulated cardioprotective signaling, AMP-activated protein kinase (AMPK), and extracellular signal-regulated kinase (ERK) signaling pathways. The ROS fluorescent probe H_2_DCFDA determination indicated that ISL significantly reduced cardiac ROS level during hypoxia/reoxygenation. Moreover, ISL reduced the mitochondrial potential (Δ*ψ*) of isolated mouse cardiomyocytes. Taken together, ISL as a natural antioxidant demonstrated the cardioprotection against ischemic injury that may attribute to the activation of AMPK and ERK signaling pathways and balance of cellular redox status.

## 1. Introduction

Myocardial infarction is one of the major causes of death in the world. Although restoration of blood flow is the only way to save the myocardium from necrosis, reperfusion-induced injury is at the background of a high mortality rate [1]. Extensive studies showed that myocardial ischemia-reperfusion (I/R) injury is associated with increased generation of reactive oxygen species (ROS). The ROS may result in depressed contractile function, arrhythmias, depletion of endogenous antioxidant network, and membrane permeability changes [2]. There is evidence that AMP-activated protein kinase (AMPK) signaling pathway is involved in cardiac redox regulation [3–6]. Our laboratory and others have provided clear evidence that AMPK plays a critical role in protection against ischemia/reperfusion injury in the heart [7–14]. 

Isoliquiritigenin (ISL), a simple chalcone-type flavonoid, is derived from licorice compounds, and present in foods, beverages, and tobacco [15]. It has been reported to possess a wide range of biological and pharmacological activities including antioxidative activity [16], antiplatelet aggregation effects [17, 18], antitumor activities [19], and estrogenic properties [20]. It has been reported that pretreatment with ISL markedly decreased the severity of reperfusion-induced arrhythmias and myocardial infarct size and reduced the activities of lactate dehydrogenase (LDH) and creatinine phosphokinase (CPK) [18]. Increased JAK2/STAT3 phosphorylation in the heart by ISL appears to the mechanism by which ISL protects the heart against ischemia and reperfusion injury [18]. To further characterize the cardioprotective effects of ISL on cardiomyocytes under hypoxic stress, we isolated mouse cardiomyocytes to investigate the effects of ISL on cardiomyocytes contractile functions during hypoxia/reoxygenation and the signaling mechanism that mediated ISL action on cardiomyocytes. 

## 2. Materials and Methods

### 2.1. Drugs and Chemicals

**Scheme 1:**
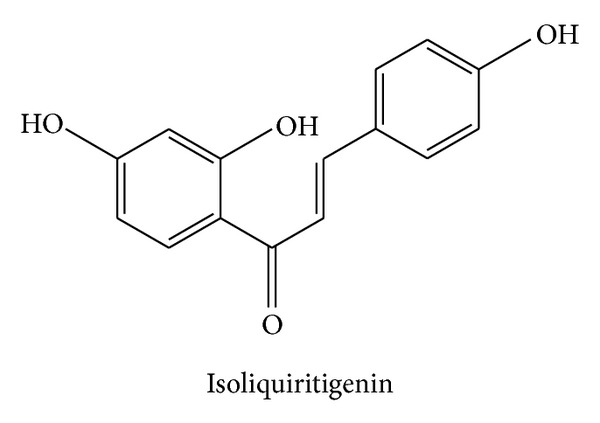


Isoliquiritigenin (ISL) was purchased from Sigma (St. Louis, MO) (Scheme 1). ISL was dissolved in dimethyl sulfoxide (DMSO) to produce a stock solution of 10 mmol/L, and the DMSO final concentration was less than 0.01% (v : v). Other chemicals were of analytical purity.

### 2.2. Animals and Cell Line

8–12-week-old male FVB/NJ mice were purchased from the Jackson Laboratory (Bar Harbor, ME, USA). All animals were kept in the institutional animal facility at the State University of New York (SUNY) at Buffalo and were fed *ad libitum*. All animal procedures used in this study were approved by the Institutional Animal Care and Use Committees at the State University of New York (SUNY) at Buffalo.

### 2.3. Isolation of Mouse Cardiomyocytes

Cardiomyocytes were enzymatically isolated as previously described [21]. Briefly, adult mouse hearts were removed and perfused with oxygenated (5% CO_2_/95% O_2_) Krebs-Henseleit bicarbonate (KHB) buffer containing (in mM) 118 NaCl, 4.7 KCl, 1.25 CaCl_2_, 1.2 MgSO_4_, 1.2 KH_2_PO_4_, 25 NaHCO_3_, 10 HEPES, and 11.1 Glucose. All the chemicals were purchased from Sigma (St. Louis, MO, USA). Hearts were then perfused with a Ca^2+^-free KHB containing Liberase Blendzyme 4 (Hoffmann-La Roche Inc., Indianapolis, IN, USA) for 15–20 min. After perfusion, left ventricles were removed and minced to disperse cardiomyocytes in Ca^2+^-free KHB buffer. Extracellular Ca^2+^ was added incrementally back to 1.25 mM. Only rod-shaped myocytes with clear edges were selected for pharmacological test and cell contractility studies. The cardiomyocytes were treated with ISL (50, 100 *μ*M) for AMPK signaling, ROS level, mitochondrial membrane potential, and glucose uptake measurements.

### 2.4. Hypoxia Treatment

Isolated mouse cardiomyocytes were subjected to two groups (normal and hypoxic groups). Hypoxic groups were kept at 37°C in a humidified sealed chamber under a humidified atmosphere of 5% CO_2_ and 95% N_2_ for 20 min. Normal groups were placed into a water-jacketed incubator at 37°C during the same period.

### 2.5. Cardiomyocytes Shortening/Relengthening Measurement

The mechanical properties of ventricular myocytes were assessed using a SoftEdge MyoCam system (IonOptix Corporation, Milton, MA, USA) [22]. In brief, left ventricular myocytes were placed in a chamber mounted on the stage of an inverted microscope (Olympus, IX-70, Center Valley, PA, USA) and incubated at 25°C with a buffer containing (in mM): 131 NaCl, 4 KCl, 1 CaCl_2_, 1 MgCl_2_, 10 Glucose, and 10 HEPES, at pH 7.4. The cells were stimulated with suprathreshold voltage at a frequency of 0.5 Hz, 3 msec. duration, using a pair of platinum wires placed on opposite sides of the chamber and connected to an electrical stimulator (FHC Inc, Brunswick, NE, USA). The myocytes being studied were displayed on a computer monitor using an IonOptix MyoCam camera. An IonOptix SoftEdge software was used to capture changes in cell length during shortening and relengthening. Cell shortening and relengthening were assessed using the following indices: peak shortening (PS), the amplitude myocytes shortened on electrical stimulation, indicative of peak ventricular contractility; time-to-PS (TPS), the duration of myocyte shortening, an indicative of systolic duration; time-to-90% relengthening (TR90), the duration to reach 90% relengthening, an indicative of diastolic duration (90% rather 100% relengthening was used to avoid noisy signal at baseline concentration); and maximal velocities of shortening/relengthening, maximal slope (derivative) of shortening and relengthening phases, indicative of maximal velocities of ventricular pressure increase/decrease. In the case of altering stimulus frequency, the steady-state contraction of myocytes was achieved (usually after the first 5-6 beats) before PS amplitude was recorded.

### 2.6. Intracellular Ca^2+^ Transient Measurements

Isolated cardiomyocytes were loaded with fura-2/AM (0.5 *μ*M) for 15 min, and fluorescence measurements were recorded with a dual-excitation fluorescence photomultiplier system (IonOptix). Myocytes were placed on an Olympus IX-70 inverted microscope and imaged through a Fluor (St. Louis, MO, USA) ×40 oil objective. Cells were exposed to light emitted by a 75 W lamp and passed through either a 360 or a 380 nm filter, while being stimulated to contract at 0.5 Hz. Fluorescence emissions were detected between 480 and 520 nm by a photomultiplier tube after first illuminating the cells at 360 nm for 0.5 sec. and then at 380 nm for the duration of the recording protocol (333 Hz sampling rate). The 360 nm excitation scan was repeated at the end of the protocol, and qualitative changes in intracellular Ca^2+^ concentration were inferred from the ratio of fura-2 fluorescence intensity at two wavelengths (360/380). Fluorescence decay time was measured as an indication of the intracellular Ca^2+^ clearing rate. Both single and biexponential curve fit equations were applied to calculate the intracellular Ca^2+^ decay constant [21].

### 2.7. Determination of Reactive Oxygen Species (ROS)

ROS were detected as previously described [23, 24]. Briefly, 5-(6)-chloromethyl-2′,7′-dichlorodihydrofluorescein diacetate (CM-H_2_DCFDA, Molecular Probes, Eugene, OR, USA) is a cell-permeant indicator for ROS that is nonfluorescent until removal of the acetate groups by intracellular esterases, and oxidation occurs within the cells. It is intracellularly deesterified and turns into highly fluorescent DCF upon oxidation. Isolated cardiomyocytes were suspended in HEPES-saline buffer and preincubation with 10 *μ*M H_2_DCFDA at 37°C for 30 min in the darkness. After cells were washed twice, fluorescence intensity was read at excitation wavelength of 485 nm and emission wavelength of 530 nm in a fluorescence plate reader (Microplate fluorometer, Spectra GEMINIXS, Molecular Device, USA). The wells containing ISL, but not H_2_DCFDA, were used as blanks. The production of ROS is expressed as fluorescence intensity in relative to untreated control cells.

### 2.8. Assessment of Mitochondrial Membrane Potential (Δ*ψ*)

The Δ*ψ* was measured using 5, 5′,6,6′-Tetrachloro-1, 1′,3,3′-tetraethylbenzimidazolocarbocyanine iodide (JC-1, Sigma, St. Louis, MO, USA). Briefly, JC-1 is a positively charged fluorescent compound which is taken up by mitochondria proportionally to the inner mitochondrial membrane potential [25]. When a critical concentration is exceeded, JC-1 monomer forms J-aggregates and becomes fluorescent red, altering the fluorescence properties of the compound. Thus, the ratio of red (J-aggregate) green (monomeric JC-1) emission is directly proportional to the mitochondrial membrane potential. Isolated cardiomyocytes were suspended in HEPES-saline buffer and preincubation with 10 *μ*M JC-1 for 10 min at 37°C. After cells were washed twice, fluorescence of each sample was read at excitation wavelength of 490 nm and emission wavelength of 530 nm and 590 nm in a fluorescence plate reader (Microplate fluorometer, Spectra GEMINIXS, Molecular Device, USA). Results in fluorescence intensity were expressed as 590 to 530 nm emission ratio. 

### 2.9. Immunoblotting Analysis and Antibodies

Immunoblotting was performed as previously described [14, 24, 26]. Isolated cardiomyocytes proteins were resolved by SDS-PAGE and transferred onto polyvinylidene difluoride membranes (Bio-Rad, Hercules, CA, USA). For reprobing, membranes were stripped with 50 mM Tris-HCl, 2% SDS, and 0.1 M *β*-mercaptoethanol (pH 6.8). Membranes were blocked with 5% nonfat milk in TBS (pH 7.4) containing 0.1% Tween-20 for 1 h and subsequently incubated with primary antibodies (1 : 1000 dilution) at 4°C for overnight. Immunoreactive bands were detected using anti-rabbit horseradish peroxidase-conjugated secondary antibodies and visualized using chemiluminescent substrate (ECL). Rabbit polyclonal antibodies against phospho-AMPK (Thr^172^), total AMPK*α*, phospho-ACC, phospho-Akt, phospho-ERK (Thr^202^/Tyr^204^), phospho-p38 MAPK (Thr^180^/Tyr^182^), phospho-STAT3, and GAPDH were purchased from Cell Signaling (Danvers, MA, USA). The densities of immunoblot bands were analyzed using a scanning densitometer (model GS-800; Bio-Rad) coupled with Bio-Rad personal computer analysis software [27–29].

### 2.10. Glucose Uptake

2-Deoxy-d-[1-^3^H] glucose accumulation in H9c2 cells was performed as previously described [30, 31]. H9c2 cells grown in 6-well plates were washed twice with serum-free DMEM and incubated with 2 mL of the same medium at 37°C for 2 h. The cells were washed 3 times with Krebs-Ringer-HEPES (KRH) buffer and incubated with 2 mL KRH buffer at 37°C for 30 min. Insulin (10 nM, Sigma, St. Louis, MO) and/or ISL (50, 100 *μ*M) were then added to H9c2. Glucose uptake was initiated by the addition of 0.1 mL KRH buffer and 2-deoxy-d-[1-^3^H] glucose (0.21 Ci/mL, GE Healthcare, Piscataway, NJ, USA) and 5 mM glucose as final concentrations. Glucose uptake was terminated by washing the cells three times with cold PBS. The cells were lysed overnight with 1 mL 0.5 M NaOH and 0.1% SDS (w/v). The radioactivity retained by the cell lysates was determined by a scintillation counter (Beckmann LC 6000IC) and normalized to protein amount measured with a Micro BCA Protein Assay Kit (Pierce Chemical, Rockford, IL, USA).

### 2.11. Statistical Analysis

For each experimental series, data are presented as mean ± SE. Statistical comparisons were made using analysis of variance (ANOVA). Differences with *P* < 0.05 were considered statistically significant.

## 3. Results

### 3.1. ISL Ameliorated Cardiomyocyte Contractile Dysfunction Induced by Hypoxia

**Figure 1:**
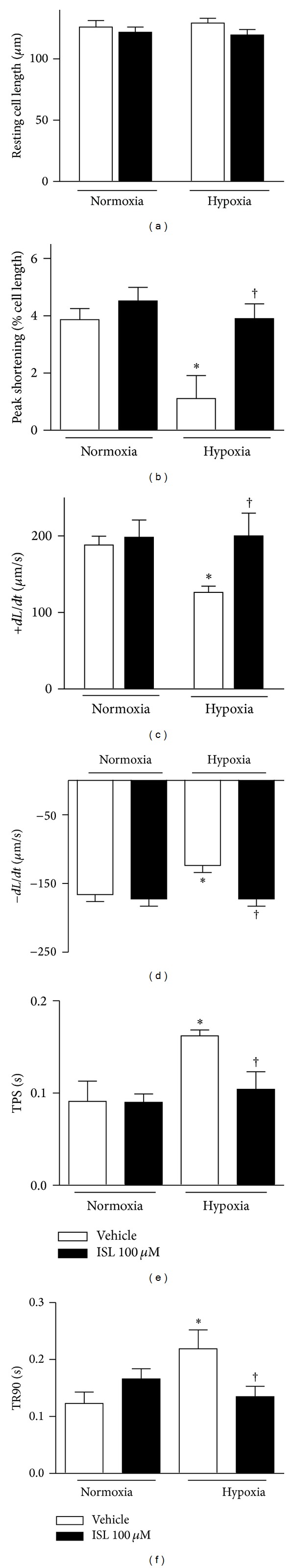
Contractile properties of cardiomyocytes from vehicle and ISL treatment after being exposed to hypoxia. (a) Resting cell length; (b) peak shortening (PS, normalized to cell length); (c) maximal velocity of shortening (+*dL*/*dt*); (d) relengthening (−*dL*/*dt*); (e) time-to-peak shortening (TPS); (f) time-to-90% relengthening (TR90). Values are means ± SE, *n* = 50–60 cells per group, *P < 0.05 versus normoxia vehicle; ^†^ *P* < 0.05 versus hypoxia vehicle.

To determine whether ISL protects cardiomyocytes against hypoxic injury, we investigated the cardiomyocyte contractility when they were exposed to hypoxia atmosphere. The mechanical properties of cardiomyocyte contractility were obtained under extracellular Ca^2+^ of 1.0 mM and a stimulus frequency of 0.5 Hz. As shown in Figure 1, ISL (100 *μ*M) treatment did not affect resting cardiomyocyte contractile function under the normal or hypoxic condition. However, during hypoxic conditions, the cardiomyocytes displayed severe impaired peak shortening (PS) (Figure 1(b)) and reduced maximal velocity of shortening/relengthening (+*dL*/*dt*, −*dL*/*dt*) (Figures 1(c) and 1(d)), while ISL treatment significantly ameliorates the contractile dysfunction of cardiomyocytes as reflected by both peak shortening and maximal velocity of shortening/relengthening (Figures 1(c) and 1(d)). Moreover, hypoxia caused the prolonged time-to-peak shortening (TPS) and time-to-90% relengthening (TR90) of cardiomyocytes (Figures 1(e) and 1(f)); however, ISL (100 *μ*M) markedly inhibited the hypoxia-induced prolonged TPS and TR90 of cardiomyocytes (Figures 1(e) and 1(f)). These results suggest that ISL protects cardiomyocytes from hypoxia-induced contractile dysfunction.

### 3.2. The Intracellular Ca^2+^ Properties of Cardiomyocytes

**Figure 2:**
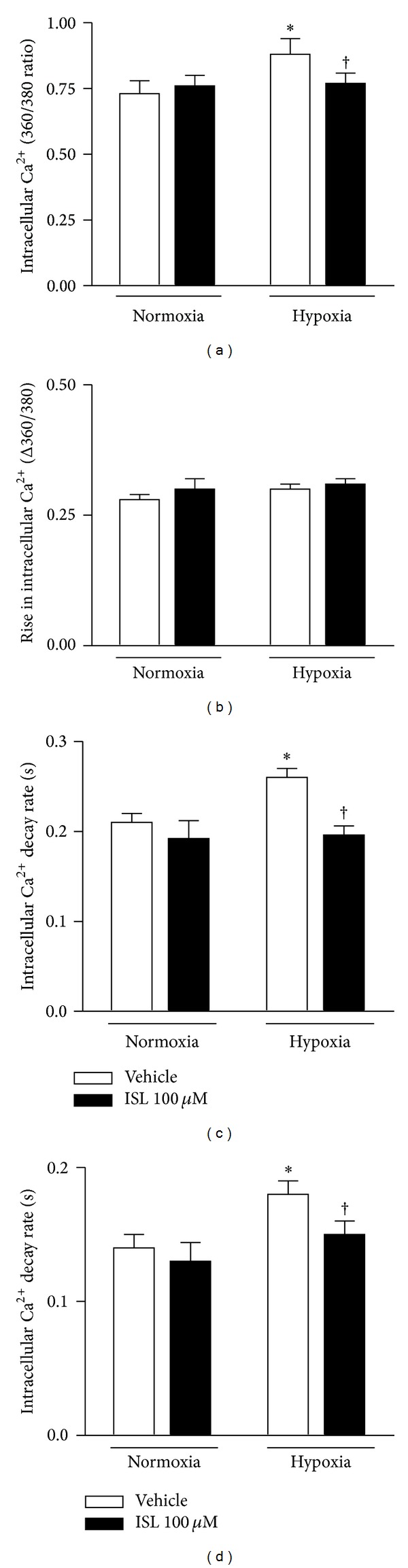
Intracellular Ca^2+^ properties of cardiomyocytes. (a) The intracellular Ca^2+^ levels; (b) the rise in intracellular Ca^2+^ levels in response to electrical stimulus; (c) the first exponential decay constant of intracellular Ca^2+^; (d) the biexponential decay constant of intracellular Ca^2+^ in response to hypoxia (20 min). Values are means ± SE, *n* = 60–90 cells per group, *P < 0.05 versus normoxia vehicle; ^†^ *P* < 0.05 versus hypoxia vehicle.

To explore the potential mechanisms involved in the protection of ISL against hypoxic cardiomyocyte contractile defect, intracellular Ca^2+^ homeostasis was evaluated using the fluorescence dye fura-2/AM [32]. The results revealed that hypoxia caused an elevation of the resting intracellular Ca^2+^ levels in isolated cardiomyocytes (Figure 2(a)) and reduced intracellular Ca^2+^ clearance with prolonging the fluorescence decay time (both single and biexponential decays, Figures 2(c) and 2(d)) as compared with cardiomyocytes under normoxia conditions. ISL (100 *μ*M) did not elicit any overt effect on resting intracellular Ca^2+^ and fluorescence decay time in nonhypoxic conditions (Figures 2(a)–2(d)), but markedly recovered the elevated resting intracellular Ca^2+^ levels and reduced intracellular Ca^2+^ clearance in isolated cardiomyocytes under hypoxic conditions (Figure 2). However, hypoxia did not change electrically stimulated rise in intracellular Ca^2+^ levels (Figure 2(b)).

### 3.3. ISL Stimulated Cardioprotective Signaling Pathways

**Figure 3:**
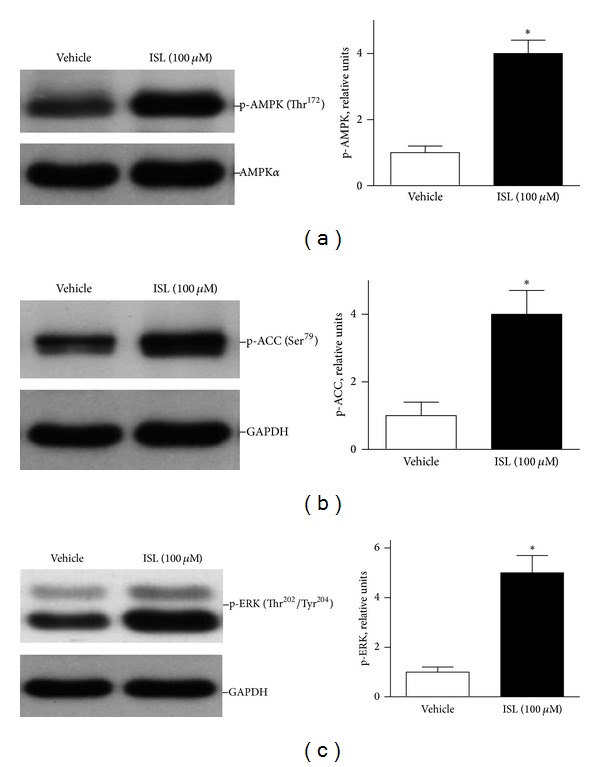
ISL treatment stimulated cardiac AMP-activated protein kinase (AMPK) and ERK signaling pathways. Representative immunoblots of isolated mouse cardiomyocytes showed phosphorylation of (a) AMPK at Thr^172^ (p-AMPK), (b) ACC (Ser^79^), and (c) ERK. Phosphorylated AMPK was quantified relative to total AMPK*α*. Phosphorylated ACC and ERK were quantified relative to GAPDH. Values are expressed as means ± SE (*n* = 3–6), *P < 0.05 versus vehicle.

Our group and others provided evidence that AMP-activated protein kinase (AMPK) is a critical signaling in cardioprotection against ischemic injury [7, 11–13]. To define the mechanism involved in the cardioprotective effect of ISL, AMPK signaling pathways were detected in isolated cardiomyocytes in response to ISL treatment. The results showed that ISL significantly triggered AMPK Thr^172^ phosphorylation as compared with vehicle group (Figure 3(a)). In parallel with AMPK activation, the downstream targets of AMPK, the phosphorylation of acetyl CoA carboxylase (ACC) was induced by ISL treatment (Figure 3(b)). Intriguingly, ISL treatment also induced extracellular signal-regulated kinase (ERK) signaling pathway in the cardiomyocytes (Figure 3(c)). These data suggest that ISL treatment can induce phosphorylation of *α* catalytic subunit at Thr^172^ of AMPK and trigger a survival signaling ERK activation.

### 3.4. ISL Decreased the Intracellular ROS Level in Isolated Cardiomyocytes

**Figure 4:**
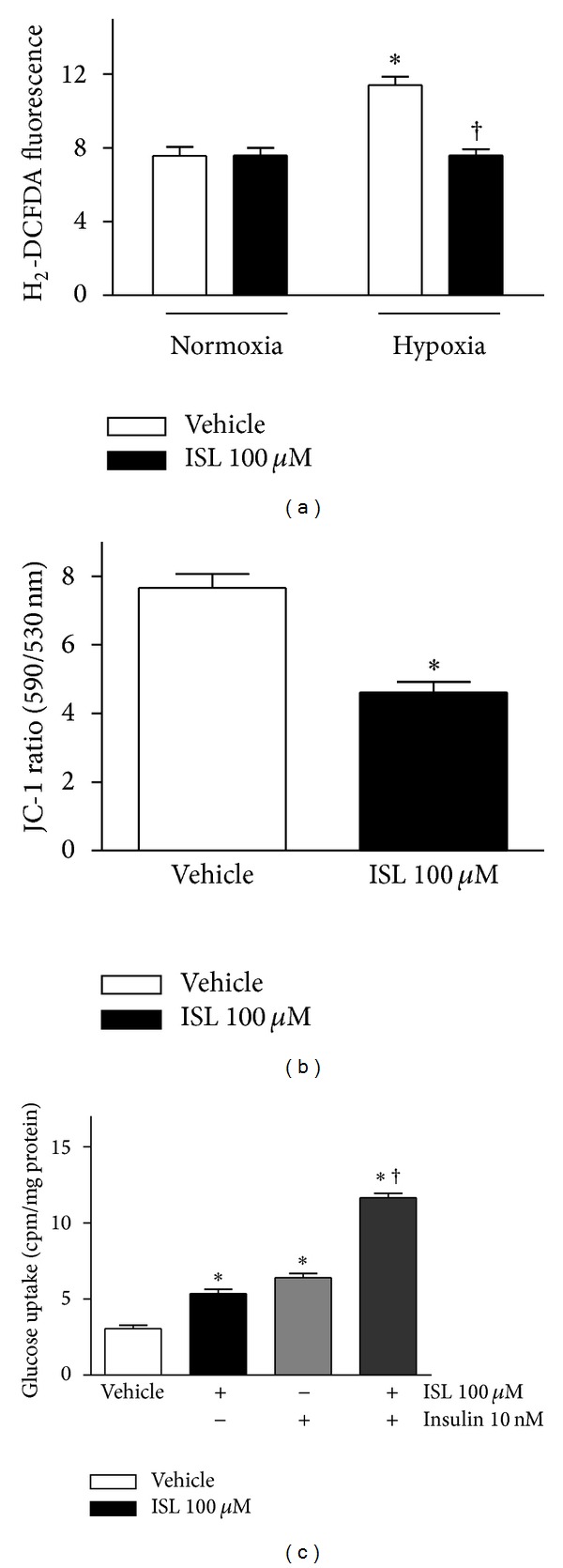
(a) ISL reduced the intracellular ROS levels in isolated mouse cardiomyocytes during hypoxia/reoxygenation. Intracellular ROS levels were measured by the fluorescent probe H_2_DCFDA after treatment with ISL (100 *μ*M) or DMSO (vehicle). ROS production was expressed as fluorescence intensity relative to untreated control cells. Data are presented as means ± SE (*n* = 4–6). *P < 0.05 versus normoxia vehicle; ^†^ *P* < 0.05 versus hypoxia vehicle; (b) ISL reduced mitochondrial membrane potential (Δ*ψ*) in isolated cardiomyocytes. Mitochondrial membrane potential (Δ*ψ*) was measured by JC-1 fluorescence assay. The result was presented as the ratio of red/green fluorescence measured at 590 nm and 530 nm, respectively. Values are means ± SE (*n* = 6–10). *P < 0.01 versus vehicle; (c) ISL treatment augmented glucose uptake of cardiomyocytes. The cardiomyocytes were preincubated for 30 min with or without ISL (100 *μ*M) and/or insulin (10 nM), before addition of 2-deoxy-[1-^3^H]glucose for additional 30 min to measure glucose uptake. Values are means ± SE for 5 experiments. *P < 0.05 versus vehicle; ^†^ *P* < 0.05 versus insulin alone.

Upon reperfusion of the myocardium after ischemia/hypoxia, there is a rapid increase in intracellular calcium that will induce the opening of the mitochondrial permeability transition pore (mPTP) [33]. Uncoupling of the electron transport chain within the mitochondria leads to the release of destructive reactive oxygen species (ROS) [34] this increase in ROS is a significant contributor to the cell death seen at the onset of reperfusion [33]. The fluorescent probe H_2_DCFDA was used to measure the effect of ISL on the level of intracellular ROS in isolated cardiomyocytes under hypoxia/reoxygenation conditions. As shown in Figure 4(a), ROS level of cardiomyocytes under hypoxia/reoxygenation was much higher than that of vehicle normoxia group (*P* < 0.01 versus vehicle normoxia). ISL treatment significantly decreased the intracellular ROS levels of isolated cardiomyocytes during hypoxia/reoxygenation (*P* < 0.05 versus vehicle hypoxia). It is suggested that ISL demonstrated cardioprotection against the hypoxia-induced contractile dysfunction through modulating the cellular redox status of cardiomyocytes.

### 3.5. ISL Reduced the Mitochondrial Membrane Potential of Cardiomyocytes

There is evidence that AMPK signaling pathway is involved in regulation of mitochondrial membrane potential (Δ*ψ*) [35]. To understand the mechanisms by which ISL activates cardiac AMPK signaling pathway, the mitochondrial membrane potential (Δ*ψ*) of cardiomyocytes was assessed using JC-1, a lipophilic fluorophore that forms J-aggregates in proportion to its intramitochondrial concentration. Isolated cardiomyocytes were preincubated for 20 min with 10 *μ*M JC-1, rinsed thoroughly, and treated with ISL accordingly.  Figure 4(b) represented the ratio of red/green fluorescence, corresponding to JC-1 in J-aggregate versus monomeric form. The results demonstrated that ISL treatment significantly reduced JC-1 dye accumulation and decreased J-aggregate formation in cardiomyocyte mitochondria in an independent manner, which indicated that ISL caused mitochondrial membrane depolarization. The Δ*ψ* reduction may contribute to the activation of AMPK induced by ISL.

### 3.6. ISL Stimulated Glucose Uptake in the Cardiomyocytes

To explore whether ISL-activated cardiac AMPK signaling modulates glucose metabolism in the cardiomyocytes, the effect of ISL on glucose uptake in cardiomyocytes was investigated using the 2-deoxy-D-1-^3^H-glucose uptake assay [31]. As shown in Figure 4(c), ISL significantly stimulated glucose uptake in the isolated cardiomyocytes (*P* < 0.01 versus vehicle). Interestingly, ISL treatment can enhance insulin-induced glucose uptake of cardiomyocytes (Figure 4(c)). 

## 4. Discussion

ROS have been implicated in the pathogenesis of stress-induced injury, including myocardial ischemia/reperfusion injury. ROS generation intracellularly contributes to contractile dysfunction and cell death during simulated ischemia/reperfusion in a perfused cardiomyocyte model [2, 36]. Extensive studies showed that some herbal extracts or active components of herbs exhibited antioxidant effects [18]. Although these studies have implicated antioxidative and cardioprotective effects of ISL, whether these actions of ISL can correlate with its cardiomyocyte contractile function is not well understood. In the present study, ISL as a natural antioxidant did not affect cardiomyocyte contractile function under the normal condition. However, cardiomyocytes displayed severe impaired contractile functions, while ISL markedly ameliorated the hypoxia-caused contractile dysfunction of cardiomyocytes. Additionally, ISL did not elicit any overt effect on resting intracellular Ca^2+^ and fluorescence decay time in nonhypoxic conditions, but it markedly elevated the electrically stimulated intracellular Ca^2+^ levels and recovered the elevated resting intracellular Ca^2+^ levels and reduced intracellular Ca^2+^ clearance in hypoxia-isolated cardiomyocytes. The explanation of mechanical defects observed in our study may be the impaired intracellular Ca^2+^ handling. The reduction of intracellular Ca^2+^ clearance is likely responsible for prolonged relaxation duration (TR90) and reduced PS in hypoxic cardiomyocytes. Meanwhile, the ROS level of hypoxic cardiomyocytes is much higher than that of normal cardiomyocytes, which may contribute to the contractile dysfunction of cardiomyocytes. When cardiomyocytes were exposed to hypoxia atmosphere, ISL treatment significantly decreased the intracellular ROS level and ameliorated the contractile dysfunction of cardiomyocytes. Generally, Hypoxia-induced ROS production may cause the membrane lipid peroxidation and protein denaturation that disturbed Ca^2+^ transportation and mitochondrial membrane potential. Therefore, the antioxidative activity of ISL could reduce the intracellular ROS levels and ameliorate the Ca^2+^ transportation and mitochondrial membrane potential; all of which contribute to the improvement of contractile function of cardiomyocytes under hypoxic stress conditions.

Currently, AMP-activated protein kinase (AMPK) pathway was revealed to be one of the signaling pathways that protect against cardiac ischemia [7, 37–39]. AMPK is a stress-sensitive kinase that can be activated by ATP depletion such as hypoxia [5], ischemia [13], and exercise [40]. Activated AMPK can phosphorylate Acetyl-CoA carboxylase (ACC) to inhibit its activity involved in fatty acid synthesis [41]. Other downstream effects of AMPK pathways include glucose uptake [42, 43], glycolysis [44], and fatty acid oxidation [45], which favor the ATP production that supply enough energy for cell living under the stress conditions. AMPK promotes glucose transport, maintains ATP stores, and prevents injury and apoptosis during ischemia [39]. Our results showed that ISL stimulated AMPK Thr^172^ phosphorylation and activation in the isolated cardiomyocytes. ISL also significantly stimulated the AMPK downstream effector glucose uptake in the cardiomyocytes. These data strongly suggest that ISL may directly trigger cardiac AMPK signaling pathway that modulates glucose homeostasis to protect hypoxia-induced cardiomyocytes injury.

AMPK has several direct molecular targets on the heart but also may interact with other stress-signaling pathways. 

On the other hand, ERK activation is antiapoptotic in most tissues [46]. Our results demonstrated that ISL as a natural antioxidant triggers ERK signaling in the isolated cardiomyocytes, even though the molecular mechanism by which ISL activates the cardiac ERK pathway needs to be characterized in future studies.

In conclusion, ISL demonstrated cardioprotection against contractile dysfunction caused by hypoxia/reoxygenation. The mechanisms of cardioprotection of ISL are associated with the activation of cardioprotective signaling pathways and modulation of intracellular redox status in the cardiomyocytes. Therefore, ISL is a potential small molecule for treatment of ischemic heart diseases in the future. In terms of our previous studies [10, 24, 27, 38, 47], regarding the clinical setting, it may be beneficial to phosphorylate AMPK during ischemic injury in patients suffering from acute myocardial infarction. For this reason, ISL could be administrated just prior to percutaneous coronary intervention (PCI) to reduce the ischemia/reperfusion injury.
